# Recovery of BDNF and CB1R in the Prefrontal Cortex Underlying Improvement of Working Memory in Prenatal DEHP-Exposed Male Rats after Aerobic Exercise

**DOI:** 10.3390/ijms21113867

**Published:** 2020-05-29

**Authors:** Dean-Chuan Wang, Hwai-Ting Lin, Yi-Ju Lee, Hsien-Fu Yu, Sin-Ru Wu, Muhammad Usama Qamar

**Affiliations:** 1 Department of Sports Medicine, Kaohsiung Medical University, Kaohsiung 807, Taiwan; whiting@kmu.edu.tw (H.-T.L.); lyr092124@gmail.com (Y.-J.L.); sjm71518056@gmail.com (H.-F.Y.); rb271880@gmail.com (S.-R.W.); usamabinqamar@gmail.com (M.U.Q.); 2 Department of Medical Research, Kaohsiung Medical University Hospital, Kaohsiung 807, Taiwan

**Keywords:** di-(2-ethylhexyl)-phthalate, working memory, brain-derived neurotrophic factor, endocannabinoids, aerobic exercise

## Abstract

Early-life exposure to di-(2-ethylhexyl)-phthalate (DEHP) has been suggested to relate to hyperactivity, lack of attention, and working memory deficits in school-age children. Brain-derived neurotrophic factor (BDNF) and endocannabinoids are induced by aerobic exercises to provide beneficial effects on brain functions. This study investigated the mechanisms underlying working memory impairment and the protective role of exercise in prenatal DEHP-exposed male rats. Sprague Dawley dams were fed with vehicle or DEHP during gestation. The male offspring were trained to exercise on a treadmill for 5 weeks, which was followed by an assessment of their working memory with a T-maze delayed non-match-to-sample task. The expressions of BDNF, dopamine D1 receptor (D1R), cannabinoid receptor 1 (CB1R), and fatty acid amide hydrolase (FAAH) in the prefrontal cortex were detected by Western blot. The results showed that DEHP-exposed rats exhibited working memory impairments without significant alterations in locomotor activities. The reduced expressions of prefrontal BDNF and CB1R were obtained in the DEHP-exposed rats, while D1R and FAAH were barely affected. Importantly, aerobic exercise during childhood-adolescence prevented the impairment of working memory in the DEHP-exposed rats by recovering the BDNF and CB1R expressions in the prefrontal cortex. These findings suggest that exercise may provide beneficial effects in ameliorating the impairment of working memory in the prenatal DEHP-exposed male rats at late adolescence.

## 1. Introduction

Plasticizer di-(2-ethylhexyl) phthalate (DEHP) exposure is ubiquitous in humans due to its widespread use in some consumer and industrial products [1]. There is a growing concern that DEHP exposure, particularly during the prenatal period, may have an impact on a child’s neurobehavioral development [2]. Prenatal DEHP exposure has been identified to alter social behavior, anxiety-like behavior, and spatial memory in young rodents [3,4,5]. In school-age children, behavioral problems such as emotional problems, aggression, hyperactivity, inattention, and working memory deficit are associated with prenatal DEHP exposure [6,7,8]. The outcome of DEHP-associated behaviors has led many researchers to notice the overlap with symptoms of attention-deficit hyperactivity disorder (ADHD) [9,10].

ADHD is characterized by inattention, hyperactivity, impulsivity, and working memory deficit [11]. Working memory refers to the ability that provides temporary storage and manipulation of the information necessary for complex cognitive tasks, which relies on the function of the prefrontal cortex [12]. The dopaminergic system exerts a strong modulating effect on working memory through the activation of dopamine D1 receptors (D1R) [13]. Several studies have reported the reduction in cortical D1R density due to various underlying conditions, i.e., neuropsychiatric disorders including working memory deficit [14,15,16]. Treatment with psychostimulant methylphenidate, a prescribed medication used to treat ADHD, can increase dopamine release and D1R density in the prefrontal cortex with improved behavioral outcomes [17,18]. Besides the involvement of D1R in working memory, a variety of experiments have focused on the role of endocannabinoids in different components of cognitive processing [19,20]. Anandamide, the most studied endocannabinoid, exerting its function through cannabinoid receptor 1 (CB1R) is degraded by the enzyme fatty acid amide hydrolase (FAAH) [21]. CB1R is expressed in high concentrations in the prefrontal cortex [22]. Chronic cannabis exposure is associated with cortical CB1R downregulation and cognitive impairment, with the most robust effects on short-term episodic and working memory in humans [23,24,25]. Exposure to cannabinoid agonists during critical periods of brain development is known to cause long-term changes in the functionality of several neurotransmitter systems in adulthood, such as the dopaminergic, glutamatergic, and noradrenergic systems [26,27,28]. These results support the notion that alterations in the endocannabinoid system during the early stage of brain development can lead to the subtle dysregulation of cognitive function.

Regular exercise is associated with several health benefits. In preadolescent children, exercise-enhanced cognitive function is observed in their executive function and attention [29,30]. One of the most investigated exercise-induced mediators is the brain-derived neurotrophic factor (BDNF). BDNF is a member of the neurotrophin family and plays a major role in neuronal survival, synaptic plasticity, learning, and memory [31]. Exercise is one of the most potent lifestyle factors increasing BDNF levels in the circulation and brain [32]. Exercise-induced BDNF secretion is associated with the improvement of anxiety, depression, and cognitive impairment in humans [33,34,35]. The neurophysiological changes induced by exercise, such as dopaminergic and noradrenergic systems, considerable overlap with the neuropathological mechanisms implicated in ADHD [36]. Exercise is suggested to be an effective adjunctive treatment for improving the symptoms of ADHD [37,38].

To date, most of the studies investigating the adverse effects of DEHP exposure on brain functions have focused on the neurodevelopment of the hypothalamus and hippocampus. Few studies have examined the effects of DEHP on the prefrontal function. Considering the critical roles of BDNF and endocannabinoids in the regulation of prefrontal function, it is very likely that the DEHP-related symptoms of ADHD may be a consequence of altered BDNF and endocannabinoid regulation in the prefrontal cortex. While aerobic exercise provides beneficial effects on brain functioning, whether aerobic exercise could prevent the symptoms of ADHD in DEHP-exposed male rats is still uncertain. The present study was thus designed to address these issues by testing whether the mechanisms underlying the prefrontal working memory were impaired in the prenatally DEHP-exposed male rats and testing for the protective effects of exercise. To test this hypothesis, prenatal DEHP-exposed male rats were trained to exercise on a treadmill during childhood-adolescence followed by the examination of their working memory and the expressions of prefrontal BDNF, D1R, CB1R, and FAAH in late-adolescence. Our findings suggest that prenatal DEHP exposure exerts a prolonged adverse effect on prefrontal working memory, which is accompanied by BDNF and CB1R downregulations, and exercise provides a protective effect on ameliorating these impairments.

## 2. Results

### 2.1. Delayed Non-Match-to-Sample Task

**Figure 1:**
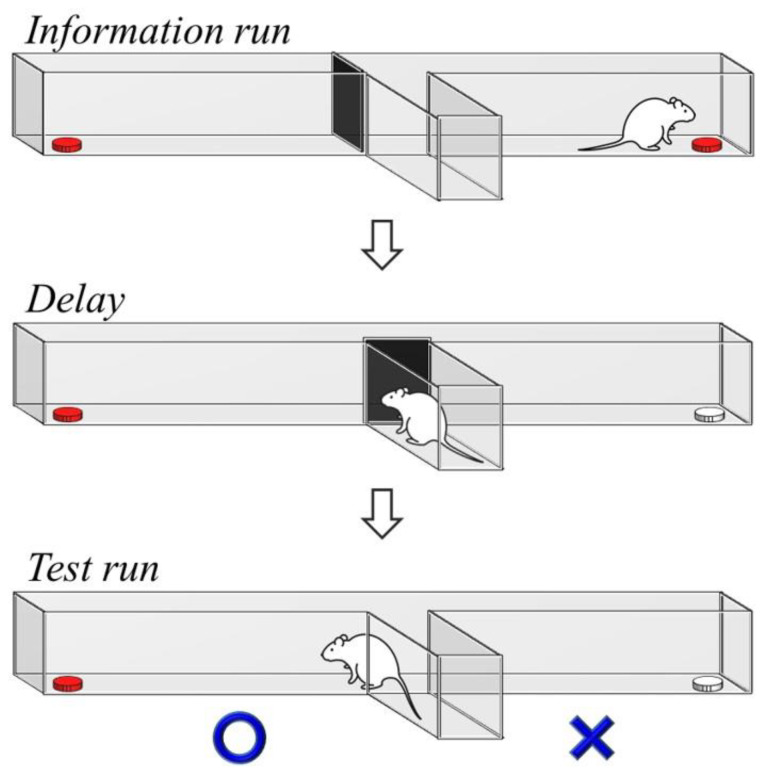
The demonstration of the delayed non-match-to-sample task. A delayed period between the information run and test run is required for the prefrontal activity to perform working memory processing. The correct and wrong choices are indicated in the test run. The white food tray represents a previously visited location in the information run.

**Figure 2:**
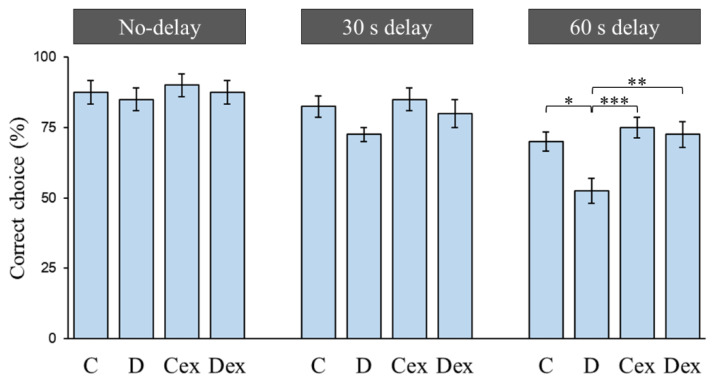
Choice accuracy in the delayed non-match-to-sample task. Animals were trained to perform the rewarded alternation in trials with no-delay, 30 s delay, and 60 s delay conditions. No significant differences were found in the no-delay and 30 s delay conditions. In the 60 s delay condition, the choice accuracy was significantly decreased in the D group. Compared with the D group, exercise improved the choice accuracy in the Dex group. C: control; D: di-(2-ethylhexyl)-phthalate (DEHP) exposure; Cex: exercised control; Dex: exercised DEHP exposure. Data are presented in mean ± SEM (*n* = 10 in each group). *: *p* < 0.05, **: *p* < 0.01, ***: *p* < 0.005.

The T-maze delayed non-match-to-sample task was applied to investigate the working memory (Figure 1). The working memory was assessed by the delayed non-match-to-sample task consisting of trials with no-delay (0 s), 30 s delay, and 60 s delay conditions (Figure 2). A two-way ANOVA revealed that there were no significant effects of treatment or exercise in the no-delay and 30 s delay conditions. In the 60 s delay condition, there were main effects of treatment (F (1, 36) = 6.128, *p* < 0.05, η^2^ = 0.15) and exercise (F (1, 36) = 9.574, *p* < 0.005, η^2^ = 0.21) on the percentage of correct choices, while no treatment × exercise interaction was found. A Bonferroni post hoc test showed that the percentage of correct choices was reduced in the D group compared to the C (*p* < 0.05), Cex (*p* < 0.005), and Dex groups (*p* < 0.01). This result suggested that prenatal DEHP exposure impaired the working memory; however, such impairments were controlled by exercise training.

### 2.2. Open Field Test

**Figure 3:**
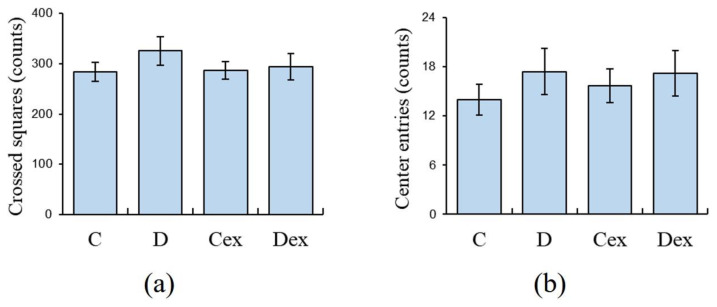
Spontaneous locomotor activities in the open field test. Animals were allowed to explore in an open field for 10 min. There were no significant differences among groups in (**a**) number of crossed squares or (**b**) center entries. C: control; D: DEHP exposure; Cex: exercised control; Dex: exercised DEHP exposure. Data are presented in mean ± SEM (*n* = 10 in each group).

The open-field test was used to investigate the locomotor activities. The two-way ANOVA revealed that there were no significant effects of treatment or exercise on either the crossed squares (Figure 3a) or center entries (Figure 3b). This result suggested that the locomotor activities in the post-adolescent male rats were barely affected by the DEHP exposure or exercise training.

### 2.3. Efficacy of Exercise Regimen

**Figure 4:**
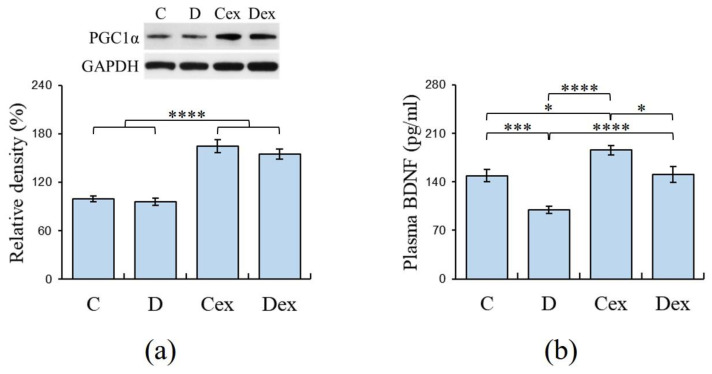
Muscle peroxisome proliferator-activated receptor gamma coactivator 1-α (PGC-1α) and plasma brain-derived neurotrophic factor (BDNF) levels analyzed by Western blot and ELISA, respectively. (**a**) Increased PGC-1α expressions were observed in the Cex and Dex groups compared to the C and D groups. (**b**) Plasma BDNF levels were significantly decreased in the D group, whereas exercise normalized these reductions in the Dex group. C: control; D: DEHP exposure; Cex: exercised control; Dex: exercised DEHP exposure. Data are presented in mean ± SEM (*n* = 10 in each group). *: *p* < 0.05, ***: *p* < 0.005, ****: *p* < 0.001.

Peroxisome proliferator-activated receptor gamma coactivator 1−α (PGC-1α) is highly expressed in the muscles after aerobic exercise to induce the mechanisms involved in muscular adaptation [39]; therefore, the expression of PGC-1α in gastrocnemius muscle was detected by Western blot to examine the efficacy of the exercise regimen. The two-way ANOVA revealed that there was a main effect of exercise (F (1, 36) = 109.155, *p* < 0.001, η^2^ = 0.75) on the expression of PGC-1α in gastrocnemius muscle (Figure 4a), while no significant effect of treatment on the expression of PGC-1α was found. The post hoc analysis showed that there was a significant increment of PGC-1α in the Cex group compared to the C (*p* < 0.001) and D groups (*p* < 0.001). Meanwhile, the increased PGC-1α was also observed in the Dex group compared to C (*p* < 0.001) and D groups (*p* < 0.001). This result suggested that the expression of PGC-1α was minimally affected by DEHP exposure and its expression was increased by aerobic exercise. This evidence supported the efficacy of the exercise regimen used in the present study.

### 2.4. Plasma Levels of BDNF

Exercise-induced BDNF protected against neuropathological diseases; therefore, the plasma BDNF levels were detected by ELISA to assess the effects of DEHP exposure and exercise on neurotrophic function. This result also provided evidence to examine the efficacy of an exercise regimen. The two-way ANOVA revealed that there were effects of treatment (F (1, 36) = 25.234, *p* < 0.001, η^2^ = 0.41) and exercise (F (1, 36) = 27.579, *p* < 0.001, η^2^ = 0.43) on the plasma BDNF levels (Figure 4b). However, there was no effect of treatment × exercise interaction on the plasma BDNF levels. The post hoc analysis showed that there was a significant reduction in plasma BDNF in the D group compared to the C (*p* < 0.005), Cex (*p* < 0.001), and Dex groups (*p* < 0.001). The result suggested that plasma BDNF was decreased by DEHP exposure, and this reduction was prevented by aerobic exercise. The efficacy of the exercise regimen was supported by the enhancement of plasma BDNF observed in the Cex group compared to the C group (*p* < 0.05).

### 2.5. Protein Levels in the Prefrontal Cortex

**Figure 5:**
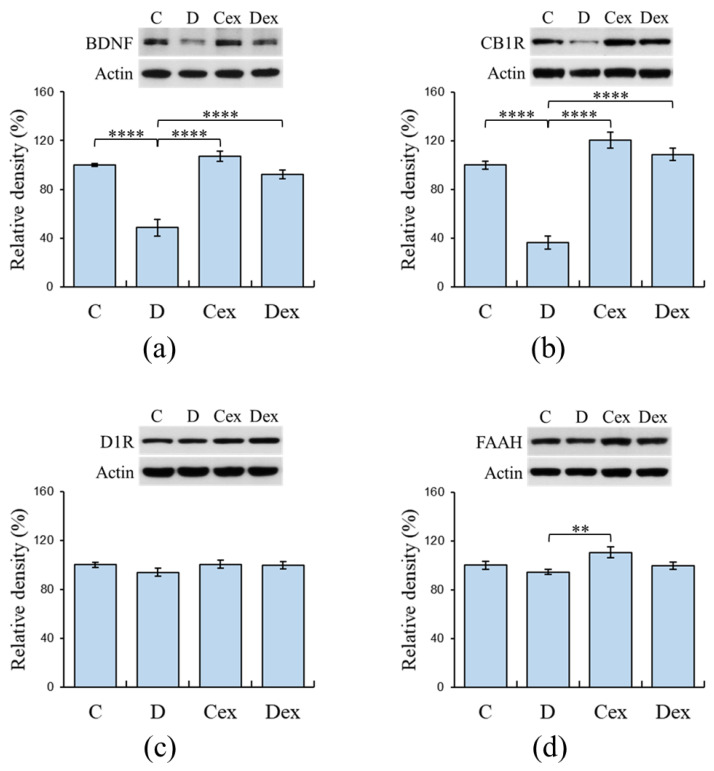
Expressions of prefrontal BDNF, dopamine D1 receptor (D1R), cannabinoid receptor 1 (CB1R), and fatty acid amide hydrolase (FAAH) analyzed by Western blot. (**a**) Decreased expressions of prefrontal BDNF were observed in the D group, whereas exercise restored this impairment in the Dex group. (**b**) Decreased expressions of prefrontal CB1R were observed in the D group, whereas exercise restored this impairment in the Dex group. (**c**) No significant differences were found among groups in their expressions of prefrontal D1R. (**d**) No significant differences were found among groups in the expressions of prefrontal FAAH. C: control; D: DEHP exposure; Cex: exercised control; Dex: exercised DEHP exposure. Data are presented in mean ± SEM (*n* = 10 in each group). **: *p* < 0.01, ****: *p* < 0.001.

The expressions of prefrontal BDNF, D1R, CB1R, and FAAH were analyzed by Western blot to examine the effects of DEHP exposure and exercise on the expressions of biomarkers related to working memory. The two-way ANOVA revealed that there were effects of treatment i.e., DEHP exposure (F (1, 36) = 53.783, *p* < 0.001, η^2^ = 0.60) and exercise (F (1, 36) = 31.949, *p* < 0.001, η^2^ = 0.47) on the prefrontal BDNF expressions (Figure 5a). A significant effect of treatment × exercise interaction on the prefrontal BDNF levels was found (F (1, 36) = 16.660, *p* < 0.001, η^2^ = 0.32). The post hoc analysis showed that there was a significant reduction in prefrontal BDNF in the D group compared to the C (*p* < 0.001), Cex (*p* < 0.001), and Dex groups (*p* < 0.001). This result suggested that prefrontal BDNF was decreased by DEHP exposure and this reduction was averted by aerobic exercise.

For prefrontal CB1R levels, the two-way ANOVA revealed that there were effects of treatment (F (1, 36) = 55.238, *p* < 0.001, η^2^ = 0.61) and exercise (F (1, 36) = 84.483, *p* < 0.001, η^2^ = 0.70) on the prefrontal CB1R expressions (Figure 5b). Additionally, the treatment × exercise interaction (F (1, 36) = 26.413, *p* < 0.001, η^2^ = 0.42) had an effect on the prefrontal CB1R expressions. The post hoc analysis showed that there was a significant reduction in prefrontal CB1R in the D group compared to the C (*p* < 0.001), Cex (*p* < 0.001), and Dex groups (*p* < 0.001). This result suggested that prefrontal CB1R was decreased by DEHP exposure and this reduction was prevented by aerobic exercise.

The two-way ANOVA revealed that there were no significant effects of treatment and exercise on the prefrontal D1R expression (Figure 5c). This result suggested that the expression of prefrontal D1R in the post-adolescent male rats were hardly affected by DEHP exposure or exercise training.

The two-way ANOVA revealed that there were effects of treatment (F (1, 36) = 6.255, *p* < 0.05, η^2^ = 0.15) and exercise (F (1, 36) = 5.533, *p* < 0.05, η^2^ = 0.13) on the prefrontal FAAH expressions (Figure 5d), while no significant treatment × exercise interaction was found. The post hoc analysis showed that there was a significant reduction in prefrontal FAAH in the D group compared to the Cex group (*p* < 0.01).

## 3. Discussion

Hyperactivity and working memory impairment are two significant symptoms in ADHD children [11]. Early-life phthalate exposure is related to the behavioral characteristics of ADHD in humans [9,10]. However, the mechanisms underlying the effects of DEHP exposure on symptoms of ADHD in the experimental animals have never been investigated. The present study showed that working memory impairment without hyperactivity symptoms was observed in the prenatally DEHP-exposed male rats. Biochemical data suggested that the DEHP-exposed rats exhibited reductions in BDNF and CB1R in the prefrontal cortex. Importantly, exercise training during childhood-adolescence reversed the impairment of working memory in the DEHP-exposed rats, which was accompanied by the recovery of BDNF and CB1R expressions in these animals.

There was no significant difference in the locomotor activity between the control and DEHP-exposed rats, suggesting that hyperactivity was absent in these animals. Previous studies have shown that the effects of DEHP on locomotor activity are inconsistent in male rodents. Neonatal DEHP exposure can induce hyperactivity [4,40], but other studies show that locomotor activities are not influenced by prenatal DEHP exposure [3,41,42]. These differences are probably caused by the doses of DEHP, age of the subjects, or time of exposure. Our data were similar to previous studies showing that locomotor activity in post-adolescent male rats was rarely affected by prenatal DEHP exposure.

The delayed non-match-to-sample task is widely used to investigate working memory in rodents. In this task, the animals are typically cued to make a choice response to obtain a reward, but are prevented from responding until after a delay period has been imposed [43]. Evidence shows that the impairment of the prefrontal cortex impairs working memory (i.e., choice accuracy) without altering reference memory (i.e., the maze task rule), suggesting that the prefrontal cortex is necessarily involved in the delayed alternation task [44]. We reported that there was no significant difference in the choice accuracy between the C and D groups under the no-delay condition, implying that prenatal DEHP exposure does not affect motivation, motor function, or reference memory for getting rewarded. There were no performance deficits at the short delay (30 s delay); however, the choice accuracy significantly decreased in the D group as the delay was extended (60 s delay). Prefrontal activity during the delay period is more important in memory retention than in inhibitory control or decision-making [45,46]. The impaired prefrontal cortex is capable of completing the correct choice at a short delay using an alternate strategy, while it exhibits a poor accuracy at longer delays as working memory demands increase [44,47]. Therefore, the DEHP-exposed rats exhibited decreased choice accuracy at longer delays, suggesting that prenatal DEHP exposure impaired working memory in the post-adolescent male rats. Currently, there is no report regarding the effect of DEHP on prefrontal working memory using delayed non-match-to-sample tasks. A study regarding the prefrontal function shows that perinatal exposure to a mixture of phthalates results in a deficit in cognitive flexibility [48]. Several studies in rodent models implicate that prenatal DEHP exposure impairs hippocampus-dependent learning and memory by enhanced oxidative damage, decreased N-methyl-d-aspartic acid (NMDA) receptors, reduced neurogenesis, and the impairment of neuronal excitability [3,41,49,50]. Although mechanisms involved in the adverse effect of DEHP exposure on hippocampal learning and memory have been identified, there is little to no evidence regarding the mechanism of working memory deficit after DEHP exposure.

Dopamine and endocannabinoids are two important regulators of prefrontal function. Evidence shows that extreme hypo- or hyper-stimulation of either D1R or CB1R impairs working memory, suggesting that appropriate levels of prefrontal D1R and CB1R are important for efficient working memory processing [14,23,24,51]. In this study, the expression of D1R was mildly affected, while the expression of CB1R was reduced in the DEHP-exposed rats. The effect of DEHP exposure on dopaminergic function has been reported by some researchers. Preadolescent DEHP exposure results in reduced midbrain tyrosine hydroxylase activity and decreased striatal D2R expression in the post-adolescent animals [52,53]. However, neonatal DEHP exposure reduces the midbrain D1R expression, but striatal D1R expression remains unchanged in the adolescent male rats [40,54]. These results suggest that the effect of DEHP exposure on dopaminergic function may depend on the region-specific susceptibility or time of exposure. Prefrontal D1R density rises dramatically at adolescence, and this upregulation of D1R is independent of gonadal hormones actions [55,56]. The anti-androgenic and estrogenic effects of DEHP may have less influence on the expression of prefrontal D1R during development, or the ceiling effect of overexpression of prefrontal D1R may have masked its detection in the present study.

Decreased prefrontal CB1R is strongly correlated with working memory disturbances in patients with schizophrenia, Parkinson’s disease, and Huntington disease [57,58,59]. Besides this, specific single nucleotide polymorphism in CB1R gene is associated with reduced prefrontal CB1R expression and working memory deficit [19,60]. Endocannabinoids act as retrograde messengers in inhibiting the release of γ-aminobutyric acid (GABA). The CB1R induced a reduction in GABA release, and GABA-mediated synaptic inhibition is impaired in the animal models of ADHD and Huntington disease [61,62]. Interestingly, evidence shows that DEHP acts as a low-affinity antagonist of CB1R [63], and DEHP exposure increases the release of GABA in the hypothalamus [64]. Based on this evidence, our result showed that reduced prefrontal CB1R may be part of the mechanisms underlying the working memory impairment caused by prenatal DEHP exposure. Additionally, excessive endocannabinoids may downregulate prefrontal CB1R and impair working memory [25]. Because anandamide is degraded by the enzyme FAAH, the alteration in FAAH expression may influence the anandamide concentration and CB1R expression. However, the prefrontal FAAH expression was not affected by DEHP exposure in the present study. Although we did not measure anandamide concentrations, our result suggested that the metabolism of anandamide was not influenced by DEHP exposure. In rodents, acute alcohol exposure reduces the prefrontal CB1R levels, while both FAAH and anandamide were not changed in the prefrontal cortex [65,66]. This inconsistency between CB1R and FAAH expressions suggests that other endocannabinoids such as 2-arachidonoylglycerol or the alteration in FAAH activity may respond to DEHP exposure.

We showed that prefrontal BDNF expressions were reduced in DEHP-exposed rats, in agreement with the reduction in hippocampal BDNF levels after DEHP exposure in male rats [67,68]. BDNF participates in the survival of neurons and promotes synaptic transmission, whereas BDNF deficiency is closely related to the pathogenesis of neuropsychiatric diseases [31]. Mice with a conditional knockout of BDNF display symptoms of ADHD [69]. Similarly, the ADHD animal model shows that there are reductions in BDNF expressions in the hippocampus and prefrontal cortex [70,71]. Besides this, the hippocampal BDNF enhances declarative and long-term memory, and the positive correlation between prefrontal BDNF and working memory has been reported [72,73]. Besides this, multiple reports have provided evidence for a crosstalk between BDNF and endocannabinoid signaling. BDNF and CB1R interactions have been demonstrated in mediating neurogenesis, neuronal survival, and protection against excitotoxicity [74]. Exogenous cannabinoids treatment increases prefrontal BDNF release [75], and the genetic deletion of CB1R decreases the hippocampal BDNF expression [76]. BDNF increases CB1R expression in cultured cerebellar granule neurons and promotes neuronal sensitivity to CB1R agonists [77]. In the present study, both BDNF and CB1R were reduced in the prefrontal cortex of DEHP-exposed rats, suggesting a plausible mechanism underlying the working memory impairment after DEHP exposure.

Exercise can enhance working memory and cognitive flexibility in humans [78,79]. The enhancement of BDNF release is the most important mechanism underlying the improvement of cognitive function after exercise [33,35]. In the present study, DEHP-exposed rats underwent exercise training for 5 weeks and exhibited an improvement in working memory and recovery of prefrontal BDNF and CB1R expressions. We noticed a major effect of exercise on increases in plasma BDNF and muscle PGC-1α, two effectors responding to aerobic exercise [33,44], providing evidence to support the efficacy of the exercise regimen used in the present study. The stimulant methylphenidate (MPH) is commonly used for ADHD treatment. A clinical study reported that, after 6 weeks of treatment with MPH, the plasma BDNF levels of ADHD children were increased and showed a significant correlation with the improvement in the symptoms of ADHD [80]. In the ADHD animal model, both exercise and MPH can increase hippocampal BDNF release and improve the symptoms of ADHD [71]. Similarly, our study showed that exercise improved the expression of BDNF in the DEHP-exposed rats, which may suggest a mechanism underlying the recovery of working memory in these rats. Exercise has been suggested as an effective adjunctive treatment for improving the symptoms of ADHD [37,38].

A study has shown that aerobic exercise increases the plasma levels of endocannabinoids in human runners [81], as well as anandamide levels and hippocampal CB1R density in rats [82]. Interestingly, there is evidence suggesting that anandamide increment during exercise may be involved in exercise-induced BDNF release and may delay the return of BDNF to basal level [83]. Exercise-induced BDNF and neurogenesis are impaired by CB1R antagonists, showing an association between CB1R activation and BDNF released during exercise [84]. Because exercise-induced BDNF and CB1R activations are dependent on exercise intensity and both effectors are involved in exercise-induced hippocampal neurogenesis, this synergism of BDNF and CB1R responding to exercise may provide more beneficial effects on improving cognitive deficits. We showed that the impaired prefrontal BDNF and CB1R expressions were recovered by exercise, suggesting that this synergism may be responsible for the recovery of working memory after exercise in DEHP-exposed rats.

In conclusion, the present study shows that prenatal DEHP exposure impairs working memory in the post-adolescent male rats. A possible mechanism underlying this finding is the downregulation of prefrontal BDNF and CB1R. Aerobic exercise during childhood-adolescence restores prefrontal BDNF and CB1R and improves working memory in DEHP-exposed rats. Therefore, because of the similarity in working memory between rats and humans, these findings may be important when investigating the ontogeny of ADHD, as well as the intervention of physical exercise in ameliorating ADHD symptoms.

## 4. Materials and Methods

### 4.1. Animals

Sprague Dawley rats (BioLasco, Taipei, Taiwan) were housed in a 12/12-h light/dark schedule (lights on at 07:00) under constant temperature and humidity. DEHP-free Altromin 1320 rat pellets (Altromin, Im Seelenkamp, Germany) and distilled water were provided ad libitum. This study was carried out in strict accordance with the recommendations of the Guide for the Care and Use of Laboratory Animals of the National Institutes of Health. All the experimental procedures were approved by the Animal Care and Use Committee of Kaohsiung Medical University (IACUC Approval Number: 106208, approved on 20 December 2017), and all efforts were made to minimize suffering and the number of animals used.

### 4.2. Experimental Design

Normal female and male rats were mated for 5 days. A vaginal plug was obtained at gestation day 1. Pregnant rats were housed individually and administered daily with corn oil (served as vehicle control, *n* = 10) or DEHP (*n* = 10) by oral gavage from gestational days 14 to 21. The pups were examined with anogenital distance on postnatal day 1, and the litters were culled to four males and four females in one cage. The offspring male rats were weaned at postnatal day (PND) 21 and then housed individually until the end of the experiment. The offspring male rats were divided into four groups: control (C, *n* = 10), DEHP (D, *n* = 10), exercised control (Cex, *n* = 10), and exercised DEHP (Dex, *n* = 10). For each group, one male sibling was taken from each litter to reduce “litter effects” [85]. In the exercised groups, the rats were trained to run on a treadmill for 5 weeks from PND 22 to PND 56. The open-field test was performed at PND 57 and the delayed non-match-to-sample task was performed from PND 58 to PND 66. The post-adolescent male rats were sacrificed on PND 67 and the samples of plasma, prefrontal cortex, and gastrocnemius muscle were isolated for further analysis. The levels of plasma BDNF were analyzed by an enzyme-linked immunosorbent assay (ELISA). The expressions of prefrontal BDNF, D1R, CB1R, FAAH, and muscle peroxisome proliferator-activated receptor gamma coactivator 1-alpha (PGC-1α) were analyzed by Western blot.

### 4.3. Gestational Administration of DEHP

DEHP (Sigma-Aldrich, St. Louis, MO, USA) was dissolved in corn oil (Sigma-Aldrich, St. Louis, MO, USA), which was prepared fresh every day. The dose of DEHP exposure was 10 mg/kg/day. The control group was supplied with corn oil, and the DEHP group was supplied with the DEHP/corn oil mixture. The estimated DEHP exposure for the adult human population is 1 to 30 µg/kg/day [86]. According to the conversion coefficient, based on the body surface area difference between humans and rats, humans are exposed to DEHP doses corresponding to 0.18–2.5 mg/kg/day for exposure in rats [87,88]. The no-observed-adverse-effect level (NOAEL) of DEHP for humans is 48 mg/kg/day, which is converted to equivalent dose corresponding to 300 mg/kg/day for rats [88]. Therefore, prenatal exposure to DEHP at the dose of 10 mg/kg/day is lower than NOAEL and considered human friendly with no known adverse effects.

### 4.4. Treadmill Running

Because rats are nocturnal animals, they were trained to run on a treadmill at night (19:00–1:00, when the light was off) to increase their motivation in running. Initially, rats in the exercise groups were allowed to run on a motor-driven horizontal treadmill (Model Exer 3/6, Columbus Instruments, Columbus, OH, USA), starting at a very low speed and gradually reaching 6 m/min for 30 min each day for 7 days to become familiar with treadmill running. Then, the animals were trained for 30 min/day, 7 days/week for 4 weeks. The running speed started at 8 m/min, increased to 11 m/min every week, and reached up to 20 m/min at the end of the training period. The rats were trained on a treadmill without electric foot shock to reduce stress during treadmill running. In contrast, animals in the non-exercising group were placed on the treadmill without running for 10 min each day for 5 weeks.

### 4.5. Open Field Test

An open field apparatus that consists of an empty square arena (60 cm length × 60 cm width) divided into 36 identical squares and surrounded by black plastic walls (40 cm height) was used. Each rat was first placed in the center of the arena and its locomotor activity was recorded for 10 min. The arena was thoroughly cleaned with 40% ethanol and allowed to dry between subjects to eliminate the possibility of any odor cues. All trials were conducted between 19:00 and 21:00 and each rat was tested only once. The spontaneous locomotor activity was determined by the total number of crossed squares and the number of entries in the central area (20 × 20 cm) (center entries). Data were recorded respectively for each rat by an observer blind to the experimental treatments. Both parameters were counted for all four paws being inside the square or the central area.

### 4.6. Delayed Non-Match-to-Sample Task

The T-maze delayed non-match-to-sample task was applied to investigate the performance of working memory, as previously reported [89]. Again, all the trials were conducted between 19:00 and 21:00. A T-maze apparatus consists of a start arm (50 cm length × 12 cm width × 25 cm height) and two-goal arms (60 cm length × 12 cm width × 25 cm height each) made from gray plastic. The crossed central area was equipped with three sliding doors. The goal arms were specified for the food reward supplied in the food trays. To increase the rats’ motivation to obtain food, they were fed with restricted amounts of chow that was sufficient to maintain each animal above 90% of its free-feeding body weight throughout the experiment. There were three steps for the delayed non-match-to-sample task: habituation, non-match-to-sample, and a delayed non-match-to-sample task. The animals underwent a habituation process from PND 58 to PND 60. The rats were allowed to explore the T-maze apparatus for 3 days (five explorations/day). During each exploration, the rats were allowed to explore freely in the T-maze and eat the food reward available at the end of both goal arms. The food reward was a piece of cereal (Quaker oats, Chicago, IL, USA) in each goal arm. After eating the cereal from both sides, the rats were returned to the start arm with the door closed for 1 min before starting another round. The T-maze was thoroughly cleaned with 40% ethanol and allowed to dry between subjects to eliminate any odor cues. After habituation, the non-match-to-sample task was performed from PND 61 to PND 65. The rats were trained to perform the rewarded alternations, in which a trial consisted of an ‘‘information run’’ and a ‘‘test run’’. In the information run, one goal arm was blocked, forcing the animal into another goal arm to get the food reward. After finishing the food reward, the block was removed and the rat was returned to the start arm to perform the test run. The rats were trained to locate the previously visited goal arm and alternate the choice to get another food reward. Rats received one food reward for choosing the previously unvisited arm (correct choice), whereas choosing the previously visited arm got no reward (wrong choice). Left/right allocations for the information and test runs were pseudo-randomized over eight trials per day, with no more than three consecutive information run to the same side. The inter-trial interval was 1 min. After 5 days of training, the percentage of correct choices >75 % was required to fulfill the criterion for evaluations. In this study, all the animals reached this criterion. Following this trial, the delayed non-match-to-sample task was performed on PND 66. The procedure of delayed non-match-to-sample task was almost identical to the rewarded alternations, except for a delayed period between the information run and test run. After finishing the information run, the rats were returned to the start arm with the door closed and stayed there for 0, 30, or 60 s in a randomized order. There were four trials for each delay and the percentages of correct choices were calculated for comparison. Data were recorded respectively for each rat by an observer blind to the experimental treatments.

### 4.7. Blood and Tissue Sample Collection

The animals were sacrificed by the inhalation of CO_2_ for 3 min. The blood samples were collected from the atria and centrifuged at 1500 rpm for 30 min, then the supernatants were collected and stored at −80 °C. After blood collection, the brain and gastrocnemius muscle were removed and soaked in an ice-cold phosphate-buffered saline solution (0.05 M Na_2_HPO_4_ and 0.137 M NaCl, pH 7.4). The whole prefrontal cortex was isolated under microscopic observation by dissecting the lateral to the forceps minor of the corpus callosum, anterior to the genu of the corpus callosum, and demarcated laterally by the accessory olfactory bulb. The muscle sample was dissected from the mid-belly region of the gastrocnemius muscle.

### 4.8. Western Blot

The tissue samples were homogenized in an ice-cold lysis buffer (20 mM Tris, pH 7.5, 150 mM NaCl, 1 mM EDTA, 1 mM EGTA, 1% Triton X-100, 1% deoxycholate, 1 mM sodium fluoride, and 2 mM sodium orthovanadate) and centrifuged at 13,000× *g* for 20 min at 4 °C. Protein in the supernatant was quantified using a BCA Protein Assay Kit (ThermoFisher, Waltham, MA, USA) according to the manufacturer’s instructions. Thirty micrograms of protein were mixed with NuPage LDS sample buffer (Invitrogen, Carlsbad, CA, USA) and separated by pre-cast 4–12% Bis-Tris gel (Invitrogen, Carlsbad, CA, USA) in MOPS running buffer (Invitrogen, Carlsbad, CA, USA) for 50 min under 120 mA and 200 V. Proteins were transferred to polyvinylidene difluoride (PVDF) membrane (Millipore, Burlington, MA, USA) in NuPage transfer buffer (Invitrogen, Carlsbad, CA, USA) for 60 min under 170 mA and 30 V condition. After blocking with 5% nonfat milk in TTBS buffer (10 mM Tris, pH 7.5, 150 mM NaCl, and 0.1% Tween 20), the membrane was incubated with primary antibodies specific for each protein for 24 h at 4 °C: rabbit anti-BDNF antibody (1:1000, ab108319, Abcam, Eugene, OR, USA), mouse anti-D1R antibody (1:200, sc-33660, Santa Cruz, Santa Cruz, CA, USA), rabbit anti-CB1R antibody (1:200, ab23703, Abcam, Eugene, OR, USA), mouse anti-FAAH antibody (1:200, sc-100739, Santa Cruz, Santa Cruz, CA, USA), mouse anti-PGC-1α antibody (1:200, ST1202, Merck, Kenilworth, NJ, USA), rabbit anti-GAPDH antibody (1:3000, ab9485, Abcam, Eugene, OR, USA), and mouse anti-actin antibody (1:5000, A2228, Sigma-Aldrich, St. Louis, MO, USA). After washing, the blot was incubated with horseradish peroxidase (HRP)-conjugated goat secondary antibodies (1:2000, ab97051 and ab97023, Abcam, Eugene, OR, USA) for 60 min at room temperature. The expression of the protein was detected by enhanced chemiluminescence (Invitrogen, Carlsbad, CA, USA) according to the recommended conditions. A Western blot analysis was performed in duplicate for each sample, and average protein levels were calculated for comparison. Digital images of the blots were created by scanning the blots, and the optical densities were determined with the Image-Pro Plus software (Media Cybernetics, Rockville, MD, USA). Each protein level was normalized to the control samples from the same membrane and presented as a percentage.

### 4.9. Enzyme-Linked Immunosorbent Assay (ELISA)

The levels of plasma BDNF were determined by commercially available assay kits optimized for small volumes, according to the manufacturer’s instructions. The detection limit of each kit for the corresponding hormones is 12 pg/mL for BDNF (ERBDNF, Invitrogen, Carlsbad, CA, USA). In brief, 100 µL of standards and diluted serum samples (dilution factors: 2× were added to wells and incubated at room temperature for 2 h with gentle shaking. Assay diluent served as a zero standard for background subtraction to construct a standard curve. The solution was then discarded and the wells were washed four times with 400 µL of wash buffer before adding 100 µL of BDNF biotinylated antibody and incubating for 1 h at room temperature. Wells were washed 4x with buffer, followed by adding 100 µL HRP-avidin to each well for 1 h at 37 °C. An amount of 100 µL TMB substrate was then added in each well for 30 min before adding the stop solution. The wells were protected from light at all times after the addition of substrate solution. The optical density of each well was measured using an automated microplate reader (PowerWave 340, Bio-Tek, Winooski, VT, USA) set to 450 nm (correction wavelength set to 550 nm) within 30 min of adding the stop solution. A standard curve was constructed by plotting the mean absorbance for each standard against the concentration to draw a best-fit curve through the points on the graph. The concentration read from the standard curve was then multiplied by the dilution factor. Each sample was measured in duplicate, and the average level from the same rat was used for comparison.

### 4.10. Statistical Analysis

The data were analyzed by a two-way ANOVA with two main effects (treatment and exercise) and possible interactions. If significant main effects or interactions were found, the Bonferroni post hoc test was performed for multiple comparisons. The statistical analysis was performed with SPSS Statistics (v. 19.0, IBM, Armonk, NY, USA). The effect size was shown by eta square (η^2^). All the values were expressed as mean ± standard error of the mean (SEM) in the figures. Significance was assumed as *p* < 0.05.

## Abbreviations

ADHD	attention-deficit hyperactivity disorder
BDNF	brain-derived neurotrophic factor
CB1R	cannabinoid receptor 1
D1R	dopamine D1 receptor
DEHP	di-(2-ethylhexyl)-phthalate
FAAH	fatty acid amide hydrolase
GABA	gamma-aminobutyric acid
PGC-1α	peroxisome proliferator-activated receptor gamma coactivator 1-alpha
